# HBV DNA integration and somatic mutations in HCC patients with HBV-HCV dual infection reveals profiles intermediate between HBV- and HCV-related HCC

**DOI:** 10.1186/s12929-024-01094-7

**Published:** 2025-01-02

**Authors:** Chiao-Ling Li, Chia-Lang Hsu, You-Yu Lin, Ming-Chih Ho, Rey-Heng Hu, Sheng-Tai Tzeng, Ya-Chun Wang, Yasuhito Tanaka, Pei-Jer Chen, Shiou-Hwei Yeh

**Affiliations:** 1 Graduate Institute of Microbiology, National Taiwan University College of Medicine, Taipei, Taiwan; 2 Department of Medical Research, National Taiwan University Hospital, Taipei, Taiwan; 3 Graduate Institute of Oncology, National Taiwan University College of Medicine, Taipei, Taiwan; 4 Graduate Institute of Medical Genomics and Proteomics, National Taiwan University College of Medicine, Taipei, Taiwan; 5 Graduate Institute of Clinical Medicine, National Taiwan University College of Medicine, Taipei, Taiwan; 6 Genome and Systems Biology Degree Program, Academia Sinica and National Taiwan University, Taipei, Taiwan; 7 Department of Surgery, National Taiwan University Hospital, Taipei, Taiwan; 8 Department of Surgery, National Taiwan University Hospital Hsinchu Branch, Hsinchu, Taiwan; 9 Department of Surgery, National Taiwan University Hospital Yunlin Branch, Yunlin, Taiwan; 10 TCM Biotech International Corp., Taipei, Taiwan; 11 Department of Gastroenterology and Hepatology, Faculty of Life Sciences, Kumamoto University, Kumamoto, Japan; 12 Department of Virology and Liver Unit, Nagoya City University Graduate School of Medical Science, Nagoya, Japan; 13 Department of Internal Medicine, National Taiwan University Hospital, Taipei, Taiwan; 14 Center of Precision Medicine, National Taiwan University, Taipei, Taiwan; 15 Department of Clinical Laboratory Sciences and Medical Biotechnology, National Taiwan University College of Medicine, Taipei, Taiwan

**Keywords:** Hepatocellular carcinoma, Hepatitis B virus, Hepatitis C virus

## Abstract

**Background:**

In regions with a high prevalence of chronic hepatitis B virus (HBV) and hepatitis C virus (HCV) infections, coinfected patients face a heightened risk of developing hepatocellular carcinoma (HCC), termed HBV/HCV-related HCC (HBCV-HCC). We aimed to investigate the contribution of preexisting chronic hepatitis B (CHB) and subsequent chronic hepatitis C (CHC) to the development of HBCV-HCC.

**Methods:**

We examined HBV’s involvement in 93 HBCV-HCC cases by analyzing HBV DNA integration as an indicator of HCC originating from HBV-infected hepatocytes, compared with 164 HBV-HCCs and 56 HCV-HCCs as controls.

**Results:**

Next generation sequencing revealed that 55% of HBCV-HCCs exhibited clonal HBV integration, which falls between the rates observed in HBV-HCCs (88%) and HCV-HCCs (7%), with similar integration patterns to HBV-HCCs. Common HCC somatic mutation analysis indicated HCV superinfection in HBCV-HCCs correlated with increased mutation rates in the telomerase reverse transcriptase (TERT) promoter and beta-catenin genes. Transcriptome analysis showed a prevalence of replicating HCV over HBV in HBCV-HCCs, with preexisting HBV exerting a proliferative role. The comparison of clinical characteristics revealed similarities between HBCV-HCC and HCV-HCC patients, including later onset for HBCV-HCC, possibly due to HCV superinfection slowing carcinogenesis. Notably, HBCV-HCCs with the same driver mutation, HBV integration at the TERT promoter, tended to develop later and showed a better prognosis post-tumor resection than HBV-HCCs.

**Conclusions:**

Our findings shed light on the interplay between preexisting CHB and subsequent CHC in elevating the risk of HBCV-HCC. These insights are crucial for understanding viral etiology-specific carcinogenesis and guiding surveillance policies for HBCV-HCC post-antiviral therapy.

**Supplementary Information:**

The online version contains supplementary material available at 10.1186/s12929-024-01094-7.

## Background

Chronic infection with hepatitis B virus (HBV) and hepatitis C virus (HCV) constitutes a major pathogenic factor for liver diseases, cirrhosis, and the development of hepatocellular carcinoma (HCC) on a global scale. In regions where chronic HBV and HCV infections are endemic, a distinct patient subgroup is coinfected by both viruses. In Taiwan, the seroprevalence of hepatitis B surface antigen (HBsAg) is 15–20% among unvaccinated individuals, a rate approximately 3 times greater than the global average [1]. On the other hand, the HCV carrier rate among individuals over 20 years old is around 4%, again surpassing the global HCV prevalence rate by 2–3 times [2]. HBV and HCV dual infection occurs in approximately 1% of the population between the ages of 30 and 65 years in Taiwan [3]. It is primarily attributed to the superinfection of HCV in individuals already infected with HBV.

Clinical investigations have consistently demonstrated a greater risk of severe liver disease in dual-infected patients than in mono-infected patients [3–5]; thus, treating these dual-infected patients has become an urgent concern. In dual-infected patients, active replicating HCV is more common than HBV [6]. Therefore, initial antiviral treatment strategies primarily focus on achieving HCV eradication. Pegylated interferon combined with ribavirin therapy effectively achieves a sustained virological response (SVR) and cures HCV infection in 70% of dual-infected patients [7]. SVR substantially reduces the long-term risk of cirrhosis and HCC [8]. Direct-acting antiviral (DAA) treatment for dual-infected patients has become even more efficacious, with a nearly 95% cure rate for HCV [9–11]. However, DAA therapy frequently triggers HBV reactivation, indicating that residual HBV may surge once HCV is eliminated from the liver [12, 13]. In line with this, follow-up studies after curative HCV treatment have reported a higher hazard ratio of 1.64 for HCC in HBV/HCV-dual-infected patients than in HCV-monoinfected individuals [14]. These findings underscore the role of preexisting HBV infection in potentiating HCC in dual-infected patients. However, the role of the interaction between HBV and HCV and the resulting oncogenic mechanisms of HBV/HCV-related HCC (HBCV-HCC) remain elusive, making it a relevant topic to explore.

In most cases, HCC originates from individual hepatocytes. These cells undergo clonal expansion in the inflammatory microenvironment and accumulate genetic aberrations over decades in the setting of chronic hepatitis, ultimately culminating in HCC. Before HCV superinfection, the liver of HBV carriers contains two groups of hepatocytes: one group infected with HBV and another free of HBV. Both cell groups are susceptible to HCV infection and can support HCV replication. Following HCV superinfection, hepatocytes segregate into two categories: one group becomes dual infected by both HBV and HCV, and the other remains mono-infected with either HCV or HBV.

Is HCV superinfection of hepatocytes already infected by HBV more prone to HCC than infection of hepatocytes without coexisting HBV exposure? Additionally, does HCC originating from these two groups of hepatocytes exhibit distinct behaviors? To address these questions, it is essential to document previous HBV infections in HBCV-HCC patients. However, most HCCs, even those caused by HBV infection alone, lack or possess very low levels of episomal HBV DNA [15, 16]. Fortunately, a biomarker, HBV DNA integration, can document previous HBV infection. HBV DNA integration is a nonobligatory event during HBV infection of hepatocytes early in HBV infection [17]. In addition, HBV clonal integration is observed within the chromosomes of infected hepatocytes in the liver of childhood or teenage HBV carriers [18]. Thus, the integrated DNA can be a genetic marker for HCC patients with previous HBV exposure. In this study, we hypothesized that HBV DNA integration could indicate HCC originating from hepatocytes infected or primed with HBV in HBV/HCV-dual infected patients. Determining the origin of HCC might elucidate the underlying carcinogenic mechanisms and contribute to the management of dual-infected hepatitis patients for antiviral therapy or HCC surveillance.

## Methods

### Sample and clinical data collection

Three hundred thirteen virus-related frozen HCC tumor tissues or genomic DNA and RNA samples were collected from National Taiwan University Hospital and Taiwan Liver Cancer Network. The etiology of HCC was determined through serological testing for HBsAg, anti-HCV, and viral titers. HBsAg(+), anti-HCV(–) patients were classified as HBV-HCC; HBsAg(–), anti-HCV(+) and/or HCV viral titer(+) patients were classified as HCV-HCC; HBsAg(+), anti-HCV(+) and/or HCV viral titer(+) HCC patients were classified as HBCV-HCC. Clinical information, including sex, age, tumor size, tumor grade, cirrhosis status, microvascular invasion status, alpha-fetoprotein (AFP) level, and outcome after tumor resection, was collected for analysis when available.

### Capture-next generation sequencing (NGS)

Capture-NGS was performed as described in our previous study [19]. Tumor genomic DNA libraries were prepared using the TruSeq Nano DNA library preparation kit (Illumina Inc, San Diego, CA, USA). To enrich targeted DNA, the genomic DNA library was hybridized with customized probes (Integrated DNA Technologies, Coralville, IA, USA) targeting HBV genomes, including genotypes B and C, as well as human genes including the promoter of telomerase reverse transcriptase (TERT, Gene ID 7015) [20], exon 3 of beta-catenin (CTNNB1, Gene ID 1499), and exon 2–11 of tumor protein p53 (TP53, Gene ID 7157). The DNA probes were designed to provide 2 × tiling coverage across the targeted sequences and were labeled with biotin to facilitate enrichment [21]. The probe sequences were listed in Supplementary Table 1. After captured by HBV or targeted gene probes, the enriched HBV-containing DNA, the TERT, CTNNB1, and TP53 sequences were processed for NGS analysis and subjected to mutation calling. The HBV-human chimera DNA sequence was analyzed to determine the breakpoint and clonality of HBV DNA integration. The junction breakpoints in HBV were illustrated using NC_003977 as the reference [22]. The integration site of HBV in the human genome was determined by aligning the human sequence in the HBV-human junction reads to the human genome [23]. Junction breakpoints in hotspot-integrated genes were illustrated based on the genome structure of TERT and lysine methyltransferase 2B (MLL4, Gene ID 9757) [24]. The clonality of integration was defined as the ratio of the detected junction sequencing depth to the sequencing depth of the glyceraldehyde-3-phosphate dehydrogenase or TP53 region. The assembly and frequencies of basal core promoter (BCP) and precore (PC) mutations in integrated HBV DNA were analyzed using only full HBV reads.

### Transcriptome analysis

Tumor RNA was prepared as a library using a TruSeq Stranded mRNA Preparation Kit (Illumina) and sequenced with the Illumina NovaSeq 6000 platform. Quality assessment of the raw reads was performed using FastQC (v0.11.9) (https://www.bioinformatics.babraham.ac.uk/projects/fastqc/), and adapter sequences were trimmed using Cutadapt (v3.0) [25]. Alignment of the qualified reads to the human reference genome GRCh38 and quantification of gene-based expression, based on the annotation of GENCODE (https://www.gencodegenes.org/), were performed using STAR (v.2.7.2) [26]. The VirTect pipeline was used to detect viral transcripts [27]. Differential expression analysis followed the procedures implemented in the R package limma [28]. Initially, raw count data were transformed into log2-counts per million values using the ‘voom’ function, which also estimates the mean–variance relationship in the data to facilitate linear modeling. After transformation, a linear model was fitted to the data for each gene, accounting for the experimental design, and empirical Bayes smoothing was applied to the standard errors to improve the reliability of the statistical inferences. The contrasts of interest were then specified, and differential expression was assessed using moderated t-statistics. A gene with a p-value < 0.05 was considered a differentially expressed gene (DEG). Principal components analysis (PCA) was conducted using the 'prcomp' function. Preranked gene set enrichment analysis (GSEA) was performed on gene sets from MSigDB (v7.4) [29] using the R package clusterProfiler [30]. Genes were ranked based on a score calculated as sign(logFC)*-log10(p value), where logFC and p-value were obtained from differential expression analysis conducted with limma. Treatment benefit scores for sorafenib and regorafenib and Sia’s immune scores and analysis of immune cell composition were calculated as described [31, 32].

### Statistical analysis

Differences in mutation rates and categorical characteristics between groups were calculated using the chi-square or Fisher’s exact tests. Age was compared using a t-test or ANOVA, depending on the comparison group. For the comparison of clinical characteristics, samples were excluded if data were missing. Recurrence-free survival (RFS) or overall survival (OS) was analyzed using Kaplan‒Meier analysis.

## Results

### HCV superinfection in chronic hepatitis B (CHB) may delay tumor development with characteristics similar to HCV-HCC

Clinical information of 313 virus-related HCCs, including 164 HBV-HCC, 56 HCV-HCC, and 93 HBCV-HCC, was compared to characterize the difference between mono-infected HCC and dual-infected HCC patients (Table 1). Compared to other HCC patients, HBV-HCC patients had an earlier age of tumor diagnosis (median age, 55.5 years vs. 59 years, P < 0.001***), larger tumor size (> 5 cm, 40% vs. 29%, P = 0.0452*), lower cirrhosis rate (37% vs. 51%, P = 0.0158*) and higher AFP levels (> = 100 ng/mL, 47% vs. 29%, P < 0.001***). Conversely, patients with HCV-HCC were older at HCC diagnosis (median age, 67 vs. 58 years, P < 0.0001***) and had higher tumor grades (grades 3–4, 52% vs. 35%, P = 0.0184*) than other HCC patients.

**Table 1:** Demographic and characteristics of viral HCCs (N = 313)

Characteristics	HBV-HCC (N = 164)	HBCV-HCC (N = 93)	HCV-HCC (N = 56)	P-value
Male	109 (66%)	61 (66%)	31 (55%)	ns
Age (Median (range))	55.5 (17–84)	63.0 (32–82)	67.0 (45–78)	HBV vs HCV, P < 0.001^##^ HBV vs HBCV, P < 0.001^##^ HCV vs HBCV, P = 0.0162^#^ HBV vs Others, P < 0.001*** HCV vs Others, P < 0.001*** HBCV vs Others, P = 0.0052**
Tumor size > 5 cm	65 (40%)	26 (28%)	17 (30%)	HBV vs Others, P = 0.0452*
Cirrhosis	61 (37%)	48 (52%)	28 (50%)	HBV vs Others, P = 0.0158*
Tumor grade 3–4	62 (38%)	27 (29%)	29 (52%)	HCV vs HBCV, P = 0.0055^#^ HCV vs Others, P = 0.0184* HBCV vs Others, P = 0.0345*
Microvascular invasion	73 (48%)	35 (38%)	25 (45%)	ns
AFP > = 100 ng/mL	75 (47%)	25 (27%)	18 (32%)	HBV vs HBCV, P = 0.0013^##^ HBV vs Others, P < 0.001*** HBCV vs Others, P = 0.0061**

ns, no significant difference

^*^, P < 0.05; **, P < 0.01; ***, P < 0.001

^#^P < 0.0167; ^##^, P < 0.0033; ^###^, P < 0.00033 in pairwise comparison by Chi-square test

HBCV-HCC patients exhibited lower tumor grade (grade 3–4: 29% vs. 42%, P = 0.0345*) and lower AFP level (≥ 100 ng/mL: 27% vs. 43%, P = 0.0061**) than mono-infected HCC patients. Regarding the similarity to HBV-HCC or HCV-HCC patients, HBCV-HCC patients had characteristics that were more similar to those of HCV-HCC patients, including smaller tumor size, higher cirrhosis rate, and lower AFP. Most intriguingly, HBCV-HCC patients had an intermediate age at HCC diagnosis between those of patients with HBV-HCC and those with HCV-HCC (median age, HBV-HCC vs. HBCV-HCC, 55.5 years vs. 63 years, P < 0.001***; HBCV-HCC vs. HCV-HCC, 63 years vs. 67 years, P = 0.0162*). Although the patients were not followed to learn the exact time of their HBV or HCV infection, epidemiological studies corresponding to the age of the patients suggest they were likely infected with HBV before their 20 s and exposed to HCV in their 20–40 s [33, 34]. Thus, these results indicate that HCV superinfection may delay HBV-induced carcinogenesis and lead to tumor characteristics more akin to those of HCV-HCC.

### Approximately half of HBCV-HCCs harbor HBV DNA integration, with an integration pattern and timing similar to that of HBV-HCCs

**Fig. 1:**
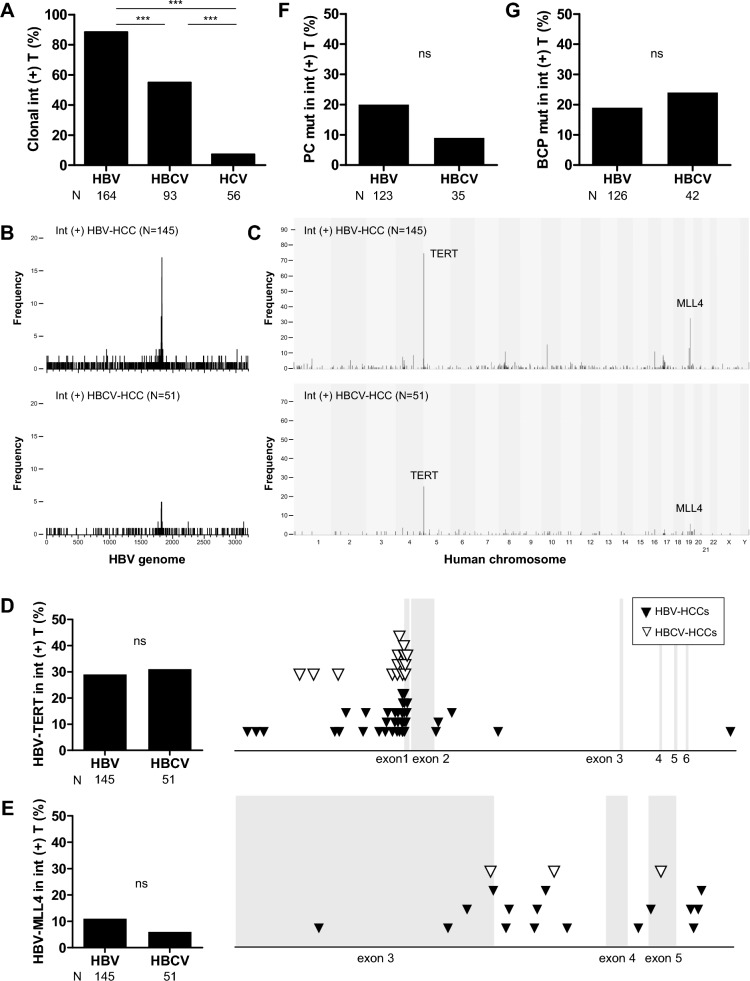
Most HBV DNA integration occurs before HBeAg seroconversion and contributes to carcinogenesis in half of HBCV-HCC cases through similar insertional mutagenesis mechanisms as in HBV-HCC. **A** Detection rate of clonal HBV DNA integration in virus-related HCCs. **B** Distribution of HBV breakpoints of integrated HBV in int(+) HBV-HCCs and int(+) HBCV-HCCs. The height of the bar indicates the frequency of junction breakpoint detected at each nucleotide in the HBV genome. **C** Distribution of HBV-human junction in the human chromosome in int(+) HBV-HCCs and int(+) HBCV-HCCs. The height of the bar indicates the frequency of junction detected in the window of 10^6^ nucleotides in the human genome. **D**, **E** Detection rates of (**D**) HBV-TERT and (**E**) HBV-MLL4 in int(+) HBV-HCCs and int(+) HBCV-HCCs (left panel) and the distribution of junction breakpoints within the indicated genes (right panel). The X-axis represents the gene position, with exon regions shaded in gray. **F**, **G** (**F**) The PC mutation rate and (**G**) BCP mutation rate for integrated HBV in int(+) HBV-HCCs and int(+) HBCV-HCCs. (***, P < 0.001; ns, no significant difference.)

To evaluate the involvement of HBV infection in the carcinogenesis of HBCV-HCC, we performed capture-NGS to identify HBV DNA integration in the tumor genome of the 313 HCCs. Capture-NGS analysis detected HBV clonal integrations in 88% of HBV-HCCs, 55% of HBCV-HCCs, and 7% of HCV-HCCs (Fig. 1A), indicating at least 55% of HBCV-HCC derived from HBV-infected hepatocytes. There was no significant difference in the number of junctions (median of junction number, integration-positive (Int(+)) HBV-HCC vs. int(+) HBCV-HCC, 4 ± 3 vs. 2 ± 4, P = 0.6300), and the distribution of junction breakpoints in HBV genome was similar between int(+) HBV-HCC and int(+) HBCV-HCC (Fig. 1B). HBV integration promotes HBV-HCC via insertional mutagenesis, cis-regulating the transcription of host genes flanking the breakpoints of viral insertion with well-reproduced hotspot genes such as TERT, MLL4, and cyclin E1 (CCNE1) [35]. The integration hotspot genes in int(+) HBV-HCCs were also identified in int(+) HBCV-HCCs (Fig. 1C), with similar detection rates and breakpoint distribution patterns, as illustrated in Fig. 1D for TERT (29% vs. 31%, P = 0.7460) and Fig. 1E for MLL4 (11% vs. 6%, P = 0.4112) (Integration in CCNE1 is not illustrated due to the low detection rates of 3.4% (5 of 145) and 2.0% (1 of 51) in int(+) HBV-HCCs and int(+) HBCV-HCCs, P = 1.0000). No novel integration hotspot specific to int(+) HBCV-HCCs was identified. Collectively, given that HBV-HCC tumors had an HBV clonal integration rate of approximately 90%, the detection of integration in 55% of HBCV-HCC cases suggests that around 60% of HBCV-HCC originated from hepatocytes previously exposed to HBV. Most clonal integrations in int(+) HBCV-HCC contribute to carcinogenesis through mechanisms similar to those in HBV-HCC, primarily through insertional mutagenesis.

We then investigated whether the timing of integration events in hepatocytes undergoing HCC transformation is similar in int(+) HBV-HCC and int(+) HBCV-HCC. HBV spontaneous nucleotide substitution rate decreases significantly once HBV is integrated since it becomes a part of the human genome [36, 37]. Therefore, the mutation of common HBV variants, PC and BCP, within integrated HBV reflects the mutation of the virion at the time of integration and can serve as markers for the timing of HBV DNA integration. The PC mutation rates were 20% and 9% (Fig. 1F, P = 0.2014), and the BCP mutation rates were 19% and 24% (Fig. 1G, P = 0.5059) for int(+) HBV-HCC and int(+) HBCV-HCC, respectively. The results indicate that the timing of integration events in hepatocytes transforming into HCC is similar in int(+) HBV-HCC and int(+) HBCV-HCC tumors. It is known that the presence of PC and BCP mutation in virion associated with hepatitis B e antigen (HBeAg) seroconversion in chronic hepatitis B (CHB) patients [38], the low PC/BCP mutation rate in clonal integrations suggest that HBV integrations in tumors mainly occur before HBeAg seroconversion.

### HCV superinfection increases TERT promoter and CTNNB1 mutation rates in HBCV-HCC regardless of HBV integration

**Fig. 2:**
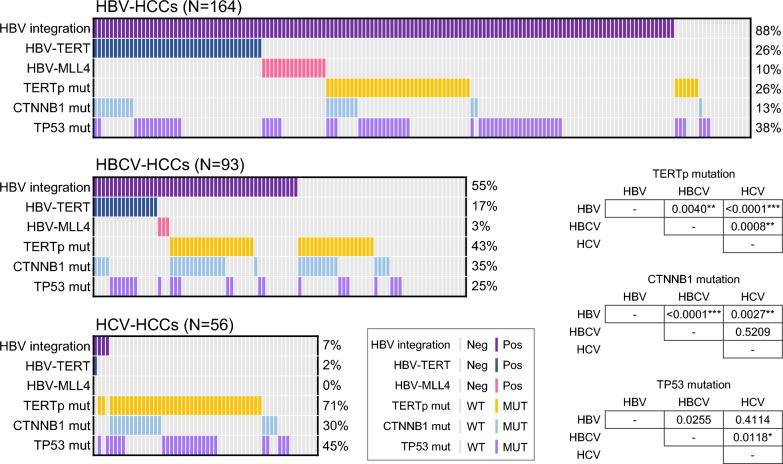
Differences in somatic mutation rates of commonly mutated genes in HCC, including the TERT promoter, CTNNB1, and TP53 in virus-related HCCs. Heatmap illustrating HBV integration and common somatic mutations detected by capture-NGS in HBV-HCCs, HBCV-HCCs, and HCV-HCCs. The positive rates for each genetic alteration are indicated on the right of the heatmap. (*, P < 0.0167; **, P < 0.0033; ***, P < 0.0003)

To further characterize the genetic difference in virus-related HCCs, we investigated the rate of the most common HCC somatic mutations, including mutations in the TERT promoter (G(−124/−146)A), CTNNB1 (exon 3), and TP53 (coding sequence), in HBCV-HCC by capture-NGS, taking HBV-HCC and HCV-HCC as reference (Fig. 2). Between HBV-HCCs and HCV-HCCs, HCV-HCCs had a significantly higher percentage of mutations in the TERT promoter (71% vs. 26%, P < 0.0001***) and CTNNB1 (30% vs. 13%, P = 0.0027**). In comparison, no significant difference was detected in the TP53 gene (45% vs. 38%, P = 0.4114) between HCV-HCCs and HBV-HCCs.

Compared to HBV-HCCs, HBCV-HCCs had significantly higher rates of TERT promoter (43% vs. 26%, P = 0.0040**) and CTNNB1 mutation (35% vs. 13%, P < 0.0001***) but a lower rate of TP53 mutation (25% vs. 38%, P = 0.0255) than HBV-HCCs. In contrast, HBCV-HCCs had significantly lower rates of TERT promoter (43% vs. 71%, P = 0.0008**) and TP53 mutation (25% vs. 45%, P = 0.0118*), while there was no significant difference in the CTNNB1 mutation rate (35% vs. 30%, P = 0.5209) when compared to HCV-HCCs. Further stratification of HBCV-HCC by the presence or absence of integrated HBV DNA did not reveal significant differences in mutation rates in the TERT promoter (41% vs. 45%, P = 0.6938), CTNNB1 (37% vs. 33%, P = 0.6941), and TP53 (29% vs. 19%, P = 0.2490). The data suggests that pre-existing HBV infection does not affect the incidence of these somatic mutations in HBCV-HCC. As a result, the elevation of mutation rate in the TERT promoter and CTNNB1 is more likely the result of HCV infection.

### RNA-sequencing (RNA-seq) results show a predominance of replicating HCV over HBV in HBCV-HCC, with preexisting HBV playing a proliferative role

**Fig. 3:**
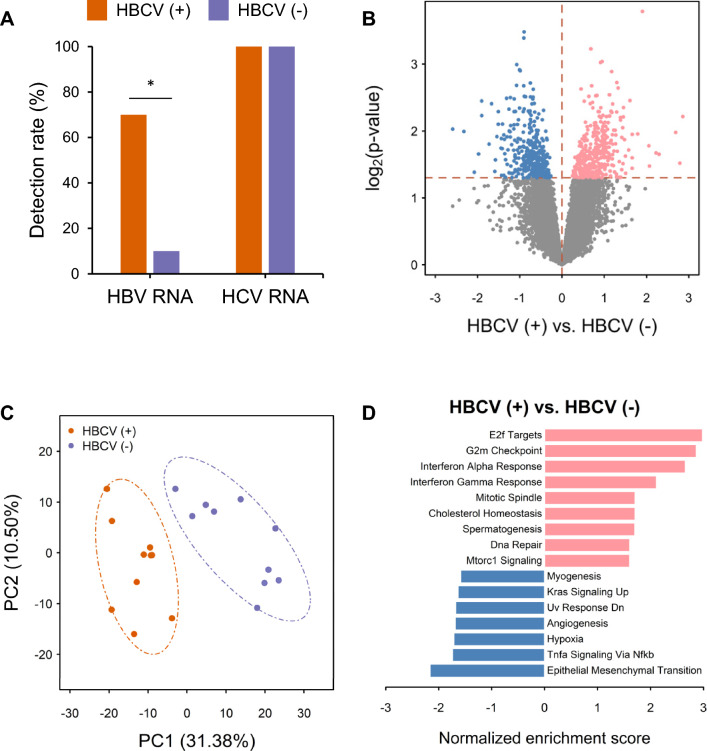
RNA-seq analysis reveals the influence of HCV and the proliferative effect of HBV on HBCV-HCC carcinogenesis. **A** Detection rate of viral transcripts in ten int(+) and ten int(–) HBCV-HCC cases. **B** DEGs between int(+) and int(–) HBCV-HCCs. **C** PCA of int(+) and int(–) HBCV-HCCs based on the DEGs in (**B**). **D** Hallmark gene sets enriched in int(+) HBCV-HCCs compared to int(–) HBCV-HCCs.

To elucidate the mechanism by which CHB contributes to the carcinogenesis of HBCV-HCC, we conducted RNA-seq analysis to profile the transcriptomes of 20 HBCV-HCC tumors, including 10 int(+) and 10 integration-negative (Int(–)) HBCV-HCC tumors. HCV RNA transcripts were identified in all (20 of 20) HBCV-HCCs, supporting the contribution of HCV to HBCV-HCC. In contrast, HBV RNA transcripts were detected in only 40% (8 of 20) of HBCV-HCCs, which was significantly lower than the detection rate of HCV transcripts (100% vs. 40%, P < 0.0001***) and more commonly identified in int(+) than int(–) HBCV-HCC (Fig. 3A, 70% vs. 10%, P = 0.0198*). The detection of HBV transcripts in 10% of int(–) HBCV-HCC is consistent with the absence of clonal integration in approximately 10% of HBV-HCC cases. Taken together, the presence of viral transcript supports the involvement of HCV in the carcinogenesis of both int(+) and int(–) HBCV-HCC, and HBV’s contribution is more common in int(+) than int(–) HBCV-HCC.

Expression profiles of int(+) and int(–) HBCV-HCCs were compared to clarify the contribution of HBV to HBCV-HCC. Differential expression analysis revealed that 370 and 369 genes were significantly upregulated in int(+) and int(-) HBCV-HCC samples, respectively (Fig. 3B). PCA was conducted on these DEGs between the int(+) and int(–) HBCV-HCC samples (Fig. 3C). GSEA suggested that proliferative hallmark gene sets, including E2F targets, G2M checkpoints, and mitotic spindles, were enriched in int(+) HBCV-HCCs (Fig. 3D), indicating that preexisting HBV confers a growth advantage to infected hepatocytes, possibly through integrated HBV DNA. Given the similarity between int(+) and int(–) HBCV-HCC in terms of common somatic mutations and clinical characteristics (Supplementary Table 2), and considering that HBCV-HCC is more similar to HCV-HCC than HBV-HCC in terms of clinical features and mutations, integration may provide a growth advantage as the basis for oncogenicity, and chronic hepatitis C (CHC) appears to be the primary driving force for carcinogenesis in the dual-infected liver due to the dominance of infection.

### HCV superinfection may delay progression of HBV-HCC and ameliorate outcomes after tumor resection

To explore clinical implications, we investigated the possible differential response to current targeted therapies for HCC of different etiology using transcriptomic analysis. Treatment benefit scores for the kinase inhibitors sorafenib [39] and regorafenib [40] were evaluated; however, no significant differences were found between HCCs (Supplementary Fig. 1A, B). In addition, gene sets related to immuno-oncology response and immune cell composition showed no significant differences between different HCC etiologies and the presence of HBV integration (Supplementary Fig. 1C, D). These preliminary results suggest no difference in tumor response to currently available systematic therapies in virus-related HCCs.

**Fig. 4:**
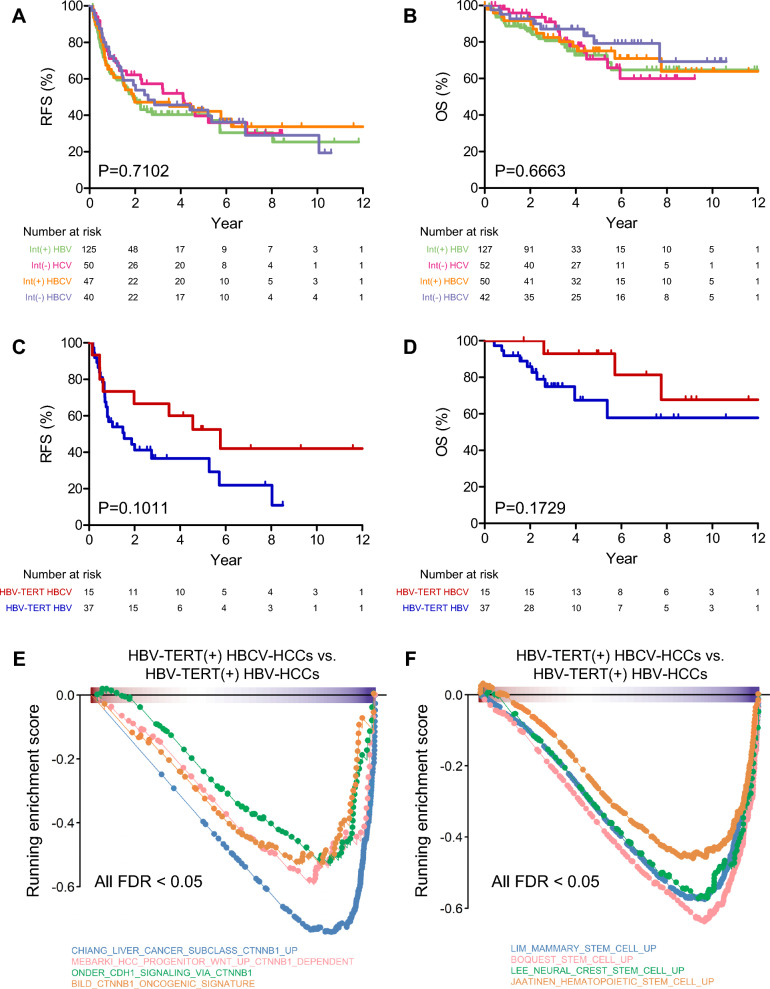
The tumor characteristics and prognosis of patients with HBV-TERT(+) HCCs tend to be mitigated by HCV superinfection. **A**, **B** Comparison of RFS (**A**) and OS (**B**) between int(+) HBV-HCCs (green line), int(–) HCV-HCCs (magenta line), int(+) HBCV-HCCs (orange line), and int(–) HBCV-HCCs (gray line) patients by Kaplan‒Meier analysis. **C**, **D** Comparison of RFS (**C**) and OS (**D**) of HBV-TERT (+) HBCV-HCCs (red line) and HBV-TERT (+) HBV-HCCs (blue line) patients by Kaplan‒Meier analysis. **E**, **F** GSEA plots of gene sets related to (**E**) CTNNB1 downstream signaling and (**F**) cell stemness between HBV-TERT(+) HBCV-HCCs (n = 3) and HBV-TERT(+) HBV-HCCs (N = 15).

Regarding the prognosis of patients with HCC after tumor resection, there were no notable differences in RFS or OS among patients with virus-related HCC, either with or without HBV integration (Fig. 4A, B). Nevertheless, to elucidate the effect of HCV superinfection in HCC, we specifically compared the clinical characteristics and prognosis between 42 HBV-HCC patients and 16 HBCV-HCC patients, all of whom use the same driver mutation, HBV-TERT, to reduce tumor heterogeneity between HBV-HCC and HBCV-HCC. Despite sharing the same driver mutation, HBCV-HCC patients had older age at tumor diagnosis (median age, 62.5 vs. 57 years, P = 0.1507), lower tumor grade (grade 3–4 tumors: 25% vs. 45%, P = 0.1594), and lower AFP levels (> = 100 ng/mL: 13% vs. 34%, P = 0.1879) than did HBV-HCC patients (Supplementary Table 3), showing the same trend as in Table 1. In addition, HBCV-HCC patients had better RFS (Fig. 4C, P = 0.1011) and OS (Fig. 4D, P = 0.1729) following tumor resection. RNA-seq showed an association of HCV superinfection with lower CTNNB1 downstream signaling (Fig. 4E) and lower stemness profiling pattern (Fig. 4F), which supports the better prognosis observed in HBV-TERT(+) HBCV-HCC patients. These findings suggest that HCV superinfection within the context of HBV infection might delay carcinogenesis, resulting in less advanced HCC and improved outcomes. However, statistical significance could not be achieved due to the limited sample size, highlighting the need for further investigation.

## Discussion

This study assesses the contributions of HBV and HCV to HCC in dual-infected individuals by analysis of virus-specific clinical characteristics, genetic alterations, and transcription. HCV superinfection generally suppresses preexisting HBV replication in dual-infected patients, disrupting HBV-induced carcinogenic mechanisms, including oncogenic HBV proteins and the microenvironment generated during CHB. Consequently, only irreversible genetic changes, such as HBV DNA integration, persist after HCV superinfection. The dominance of HCV shifts HBCV-HCC towards HCV-HCC in terms of somatic mutations and clinical characteristics.

The intriguing discovery of delayed tumor diagnosis in dual-infected patients relative to HBV mono-infected patients suggests that dual infection does not simply promote carcinogenesis synergistically, which is also observed in the previous study [3]. While suppressing CHB-induced carcinogenesis by inhibiting HBV replication, CHC creates its own inflammatory environment. In addition to the carcinogenesis of HCV-infected hepatocytes, it also fuels the clonal expansion of HBV-infected hepatocytes, which may have already acquired growth advantage during CHB before HCV superinfection. The findings explain the increased incidence of HCC in dual-infected individuals compared to mono-infected individuals. Meanwhile, the similarities in clinical characteristics and genetic mutations in int(+) and int(–) HBCV-HCC are consistent with the idea that the malignant transformation of hepatocytes in the dual-infected liver is mainly driven by CHC.

We further addressed the possible contribution of HCV superinfection by comparing HBV-HCC and HBCV-HCC with the same driver integration. HCV superinfection was associated with later tumor diagnosis, smaller tumor size, lower tumor grade, and even better outcomes. Together, dual infection increased the risk of HCC more than HBV mono-infection but delayed HCC onset and might ameliorate tumor characteristics. However, the sample size was limited, and further validation of this observation is warranted.

Notably, our study revealed the presence of clonal HBV integration in 7% of the HCV-HCC patients in Taiwan (Fig. 1A), possibly a result of resolved HBV infection. Consistently, no clonal HBV integration was detected in HCC from 12 HCV patients who had resolved HBV infection and developed HCC after achieving HCV-SVR through early DAA treatment in another cohort. The low detection rate of clonal junctions in HCV-HCC patients can be attributed to the rapid clearance of acute HBV infection and the natural elimination of int(+) hepatocytes without chronic inflammation. Nevertheless, clonal HBV integration in HCV-HCC may not merely be a remnant of previous HBV infection, as HBV-TERT was detected in one of the four int(+) HCV-HCC cases in the current study. This observation suggests that resolved HBV may contribute to carcinogenesis through residual HBV DNA integration. In view of this, early antiviral treatment after both HBV and HCV infection is crucial for reducing HBV integration events, alleviating chronic infection-induced selective environments, and collectively mitigating the risk of HCC in HBV/HCV-endemic regions.

Our previous study found that the viral-host chimera DNA (Vh-DNA) generated by HBV integration can serve as a unique circulating DNA biomarker for ~ 90% of HBV-HCC patients [41]. Therefore, clonal expansion of HBV-integrated hepatocytes in dual-infected patients or hepatitis B core antibody-positive HCV-infected patients after DAA treatment can be monitored by detecting circulating vh-DNA, which can help guide early therapy for preventing tumor development.

## Conclusions

This study elucidates the complex relationship between HBV and HCV infections and their distinct contributions to hepatocarcinogenesis of HBCV-HCC. It explains the pivotal role of HBV integration in tumor development and the influence of HCV superinfection on carcinogenesis. Our findings raise the importance of treating or closely monitoring chronic dual-infected patients after curative treatment, as they represent a high-risk group for HCC development.

## Supplementary Information

Additional file 1

Additional file 2

## Data Availability

The datasets used and analyzed during the current study are available from the corresponding author upon reasonable request.

## Abbreviations

AFP: Alpha-fetoprotein
BCP: Basal core promoter
CCNE1: Cyclin E1
CHB: Chronic hepatitis B
CHC: Chronic hepatitis C
CTNNB1: Beta-catenin
DAA: Direct-acting antiviral
DEG: Differentially expressed gene
GSEA: Gene set enrichment analysis
HBV: Hepatitis B virus
HBCV-HCC: HBV/HCV-related HCC
HBeAg: Hepatitis B e antigen
HBsAg: Hepatitis B surface antigen
HCC: Hepatocellular carcinoma
HCV: Hepatitis C virus
Int(–): Integration-negative
Int(+): Integration-positive
MLL4: Lysine methyltransferase 2B
NGS: Next generation sequencing
OS: Overall survival
PC: Precore
PCA: Principal components analysis
RFS: Recurrence-free survival
RNA-seq: RNA-sequencing
SVR: Sustained virological response
TERT: Telomerase reverse transcriptase
TP53: Tumor protein p53
Vh-DNA: Virus-host chimera DNA
