# Supplementary material for: HBV DNA integration and somatic mutations in HCC patients with HBV-HCV dual infection reveals profiles intermediate between HBV- and HCV-related HCC
Source: J Biomed Sci. 2025 Jan 2;32:2. doi: 10.1186/s12929-024-01094-7 (PMC11694426; doi:10.1186/s12929-024-01094-7)
Supplement: Supplementary file 1 — Additional file 1 [file 12929_2024_1094_MOESM1_ESM.pdf]

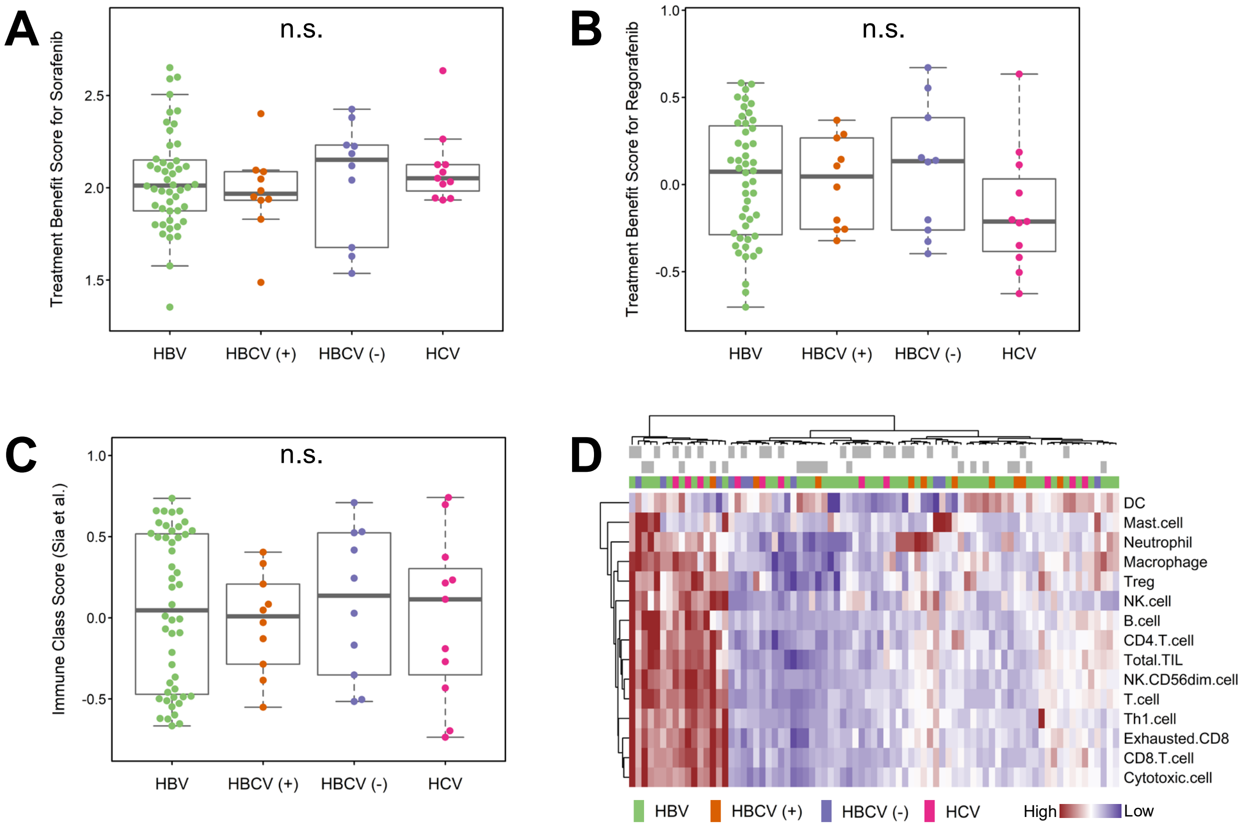


**Supplementary Figure 1. No significant differences were found in response to systematic therapies in HCC with different viral etiologies.** (A) Treatment benefit score for sorafenib in the four groups of HCCs. (B) Treatment benefit score for regorafenib in the four groups of HCCs. (C) Immune Class Score for the four groups of HCCs. (D) Immune cell expression according to Danaher's gene sets among HBV-HCC subgroups.
